# Evaluation of Japanese instructor experiences in the first overseas BLS course certified by the Japanese association for acute medicine in Cambodia: a mixed-methods analysis using text mining

**DOI:** 10.3389/fmed.2025.1679781

**Published:** 2026-01-05

**Authors:** Yoshitaka Ooya, Shuji Takahira, Kennichiro Sonoda, Rie Kiyosumi, Emiri Toyama, Nao Komuro

**Affiliations:** 1 Saitama Ika Daigaku Kokusai Iryo Center, Hidaka, Japan; 2 Department of Emergency and Acute Medicine, Saitama Medical University, Saitama, Japan; 3 Saitama Ika Daigaku Sogo Iryo Center, Kawagoe, Japan; 4 Sayama Renal Clinic, Saitama, Japan

**Keywords:** basic life support, Cambodia, communication barriers, cross-cultural medical education, instructor experience, text mining, low- and middle-income countries (LMICs)

## Abstract

**Background:**

Emergency medical education in low- and middle-income countries (LMICs) faces significant challenges, including limited resources and cultural-linguistic barriers. Understanding instructor experiences in international settings is crucial for effective capacity building.

**Objectives:**

This study examined Japanese instructors’ experiences teaching the first overseas Basic Life Support (BLS) course certified by the Japanese Association for Acute Medicine in Cambodia, exploring teaching challenges and adaptations in cross-cultural contexts.

**Methods:**

Six JAAM BLS-certified instructors participated in this exploratory mixed-methods pilot study conducted in Siem Reap, Cambodia, in March 2024. Instructors completed Visual Analogue Scale (VAS) assessments measuring accomplishment, communication clarity, instructional confidence, and cultural sensitivity, alongside open-ended questionnaires. Qualitative data underwent text mining analysis, including word frequency calculation, co-occurrence network construction, and manual sentiment classification.

**Results:**

Visual Analogue Scale scores revealed high satisfaction (sense of fulfillment: 9.1 ± 0.80; willingness to teach again: 9.2 ± 0.68) but significant communication difficulties (3.5 ± 1.52). Text mining identified three primary themes: communication challenges via interpreters, AED instruction difficulties due to English-only labeling, and practical adaptation under resource constraints. Despite challenges, instructors reported positive emotional growth through reflective practice and adaptive teaching strategies. Peer learning among participants emerged spontaneously during practical sessions.

**Conclusion:**

While language barriers and equipment shortages affected instructional clarity in cross-cultural BLS education, instructors demonstrated adaptive expertise and professional growth through reflective learning. Findings suggest that visual communication enhancement, simplified terminology, culturally adapted materials, and effective interpreter collaboration are essential for sustainable international BLS programs in LMICs.

## 1 Introduction

The World Health Organization (WHO) emphasizes developing emergency medical systems as a global health priority (1). In low- and middle-income countries (LMICs), pre-hospital infrastructure and healthcare worker training are particularly limited (2, 3). Cambodia exemplifies these challenges due to post-conflict healthcare infrastructure and urban-rural disparities (4).

As international health cooperation expands, effective health workforce development in LMICs grows increasingly important (5). A WHO Special Programme for Research and Training in Tropical Diseases evaluation of educational programs in eight countries reported insufficient training opportunities, unequal access, and competency gaps in LMICs (5). Training instructors who can teach across cultural and linguistic barriers is critically important in international emergency medical education.

Recent health professions education developments have produced educational theories addressing these challenges. Adult learning theory emphasizes learner autonomy and experiential learning, suggesting flexible teaching methods for diverse cultural backgrounds (6). Cultural humility emphasizes instructor self-reflection and openness toward different values and contexts (7). Adaptive expertise theory emphasizes flexibly adjusting teaching methods and problem-solving in resource-limited, unfamiliar environments (8).

Basic life support (BLS) education is critical to emergency medical systems, yet LMIC educational environments often face resource shortages and linguistic and cultural barriers (9–11). Organizing these challenges through educational theory provides systematic understanding of how instructors learn, adapt, and develop expertise.

In March 2024, the Japanese Association for Acute Medicine (JAAM) held its inaugural overseas BLS course in Siem Reap, Cambodia. This study explores instructor-recognized challenges and teaching adaptations, deriving implications for international instructor development through contemporary medical education theory.

## 2 Methods

### 2.1 Study design

This exploratory pilot study targeted Japanese instructors who participated in a JAAM-certified BLS course in Siem Reap, Cambodia, in March 2024. The study employed mixed-methods analysis with a small sample to identify characteristics and challenges of teaching in an international educational setting.

Course overview: Four 150-min sessions were conducted in a three-station format. Each session included the following: an introduction (60 min), CPR (35 min), AED (15 min), CPR + AED (30 min), and a wrap-up (10 min). The detailed course timetable is available in Supplementary Table 1.

### 2.2 Participants and setting

Six JAAM BLS-certified instructors taught local healthcare professionals and students through interpreter-facilitated lectures and hands-on instruction. Cambodia’s limited medical infrastructure and educational resources created constraints on teaching materials and equipment, along with linguistic and cultural differences.

### 2.3 Survey items (Visual Analogue Scale and free-response)

After the course, participating instructors completed a voluntary questionnaire.

#### 2.3.1 Survey content

##### 2.3.1.1 VAS (Visual Analogue Scale)

A 10-point scale measured sense of accomplishment, communication clarity, instructional confidence, and cultural sensitivity (0 = “not at all”; 10 = “very much”). VAS scores were calculated as mean ± standard deviation and range. Due to the small sample, no statistical tests were performed; analysis was limited to exploratory descriptive statistics.

##### 2.3.1.2 Free-response comments

Participants provided open-ended written responses regarding educational insights, challenges, and improvement areas.

### 2.4 Text mining analysis

Free-response data underwent stepwise preprocessing and natural language processing. Japanese responses were translated into English (see translation procedure below). Preprocessing involved tokenizing words using Janome tokenizer and removing stop words. Part-of-speech filtering focused on nouns, verbs, and adjectives. Word frequency was calculated using Pandas, and high-frequency words were extracted. A co-occurrence network was constructed using NetworkX to reveal word relationships, defining co-occurrence as “word combinations appearing simultaneously within the same sentence,” and visualized using Matplotlib. For emotional tone classification, dictionary-based automatic sentiment analysis limitations were recognized due to short, translated sentences and small data volume. Therefore, the research team manually classified terms into “Positive,” “Negative,” and “Neutral” categories based on context.

### 2.5 Translation procedure

Open-ended responses were machine-translated from Japanese to English. The research team reviewed translations for contextual fidelity, semantic consistency, and medical education terminology appropriateness. Where discrepancies arose, consensus review through discussion finalized translations. Recognizing that translation can influence lexical choices and interpretation (12), we established multiple-reviewer and consensus-building processes to minimize translation bias and ensure qualitative analysis reliability.

### 2.6 Ethical considerations

This study used only anonymized data without collecting personal information. Participation was voluntary; survey responses constituted research consent.

## 3 Results

### 3.1 Quantitative results (VAS evaluation)

The VAS evaluation results from six instructors are shown in Table 1. VAS scores are presented as the mean ± standard deviation (SD), range (maximum value−minimum value). Overall satisfaction with the educational experience was high, with high scores for “sense of fulfillment” (9.1 ± 0.80, 8.0−10), “willingness to teach again” (9.2 ± 0.68, 8.4−10), and “awareness of cultural differences” (9.2 ± 0.54, 8.7−10). Conversely, the “no communication problems” item scored low (3.5 ± 1.52, 1.4−5.1), suggesting communication difficulties during instruction mediated by interpreters.

**TABLE 1:** Visual analog scale (VAS) score results by item.

VAS survey content	Average scores (SD) (range)
Was the training content appropriate?	7.0 (1.3) (4.4–8.2)
Were the participants proactive?	7.4 (0.45) (7.0–8.2)
Was the training duration appropriate?	8.6 (1.4) (5.8–10)
Was chest compression training well-conducted?	6.7 (1.3) (5.1–8.9)
Was artificial respiration training well-conducted?	5.3 (0.91) (3.6–6.3)
Was AED training well-conducted?	4.8 (1.5) (2.6–6.0)
Was communication problem-free?	3.5 (1.5) (1.4–5.1)
Were the differences in social backgrounds noticeable?	9.2 (0.54) (8.7–10)
Did you feel a sense of fulfillment?	9.1 (0.80) (8.0–10)
Would you like to do it again?	9.2 (0.68) (8.4–10)

Mean visual analog scale (VAS) scores (range: 0–10) for various aspects of the instructional experience reported by the six instructors. The highest ratings were seen for “sense of fulfillment,” whereas the lowest ratings were related to communication and teaching Automated External Defibrillator (AED) and ventilation.

Regarding specific skill instruction items, “Automated External Defibrillator (AED) instruction” (4.8 ± 1.5, 2.6−6.0) and “ventilation instruction” (5.3 ± 0.91, 3.6−6.3) received relatively low scores, indicating challenges in conveying practical skills and confirming participants’ understanding.

### 3.2 Qualitative results: text mining

**FIGURE 1:**
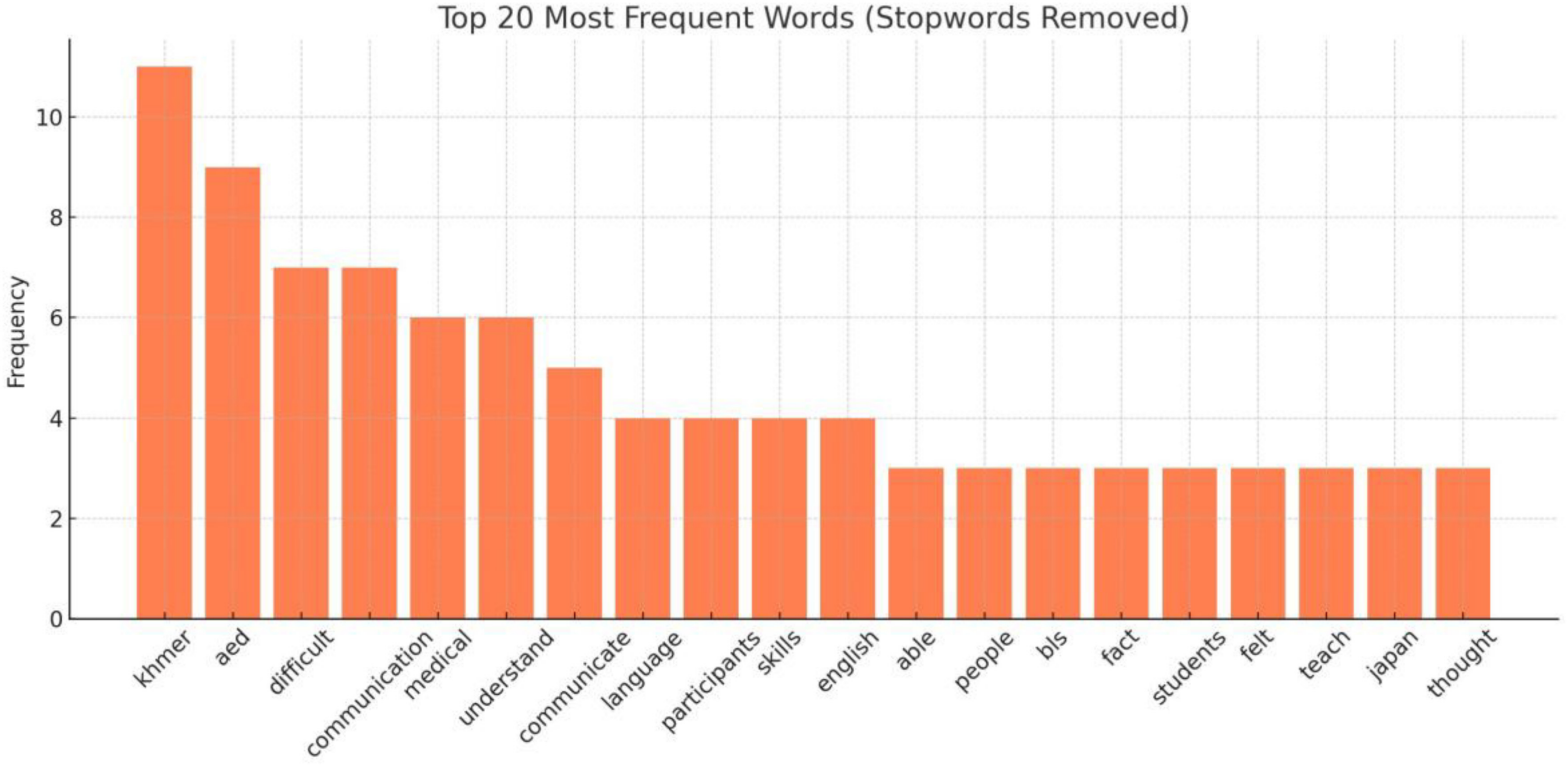
Top 20 most frequent words in instructor reflections. A bar chart displaying the 20 most frequently occurring words extracted from the free-text responses of instructors via text mining. Common terms include “communicate,” “teach,” and “understand,” highlighting key instructional and linguistic challenges.

**FIGURE 2:**
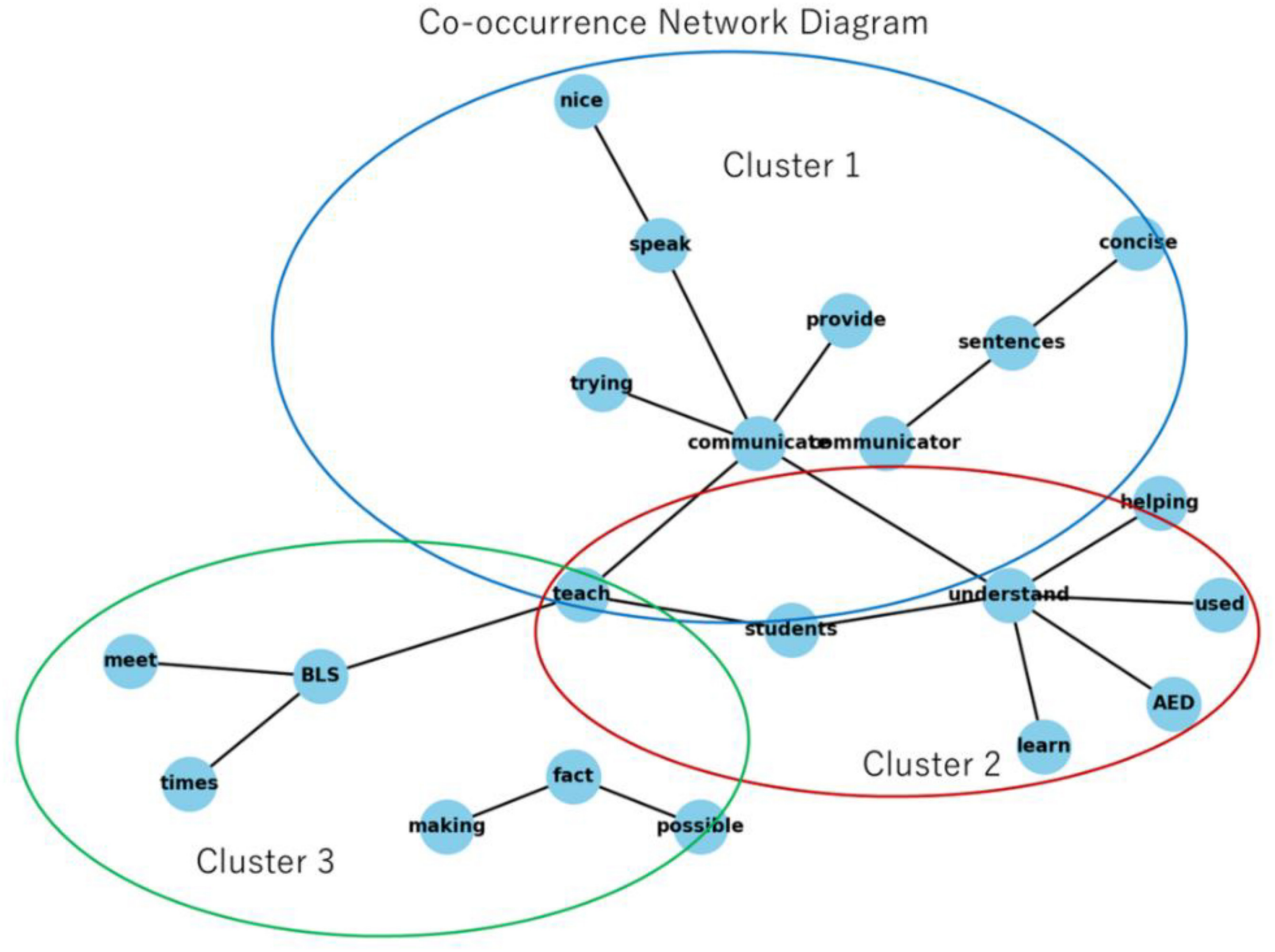
Co-occurrence network diagram of instructor comments. Network diagram based on co-occurrence analysis of instructor comments. Three main clusters emerged: Cluster 1 (blue): communication and instruction. Cluster 2 (red): AED and learning. Cluster 3 (green): BLS and practical adaptation. Key terms such as “communicate,” “understand,” “teach,” and “AED” reveal the central themes that instructors focused on in their reflections.

Analysis of open-ended responses (Table 2) revealed three primary thematic domains from word frequency patterns and co-occurrence structures. Figure 1 illustrates the top 20 frequent terms, while Figure 2 presents a co-occurrence network diagram the lexical landscape.

**TABLE 2:** Open-ended questionnaire results.

Translated comment
When communicating through an interpreter who was not a healthcare professional, I felt the need to speak in language that was easy for the interpreter to translate.
I realized the importance of speaking in short, concise sentences rather than long ones.
As an instructor, I felt I needed to learn how to adjust my communication style when teaching participants from a foreign country.
Only one AED device had Khmer-language guidance, so having more Khmer-compatible AEDs would be ideal.
It was sometimes unclear how accurately the interpreter understood and conveyed my message.
There were moments when I felt that the explanation did not get through even with interpretation, suggesting the need for strategies when communicating via an interpreter.
Cross-cultural interaction was meaningful.
Although Cambodia does not yet have AEDs, the participants were highly motivated.
Some participants were not healthcare workers, yet they were enthusiastic despite the topic being unrelated to their usual work or daily life.
I felt that such uniformly positive engagement would be rare in Japan.
Participants often taught and corrected each other during practice sessions.
Coming from a Japanese cultural background where modesty is common, I was reminded of the importance of active participation in learning.
Overall, the experience was meaningful.
Language proficiency (Khmer and English) is necessary.
Adaptability is important.
Khmer-language teaching materials are needed.
Since many participants have economic constraints, collecting course fees may be difficult.
Communication was challenging.
There was only one Khmer-language AED.
There is no medical system to provide post–cardiac arrest care even if AED use is successful.
It was challenging to transport equipment through the airport.
I was able to enjoy sightseeing.
Although our contribution to Cambodian healthcare is limited, the experience still felt like international cooperation.
To continue future programs, instructors need at least basic English communication skills and an understanding of the Cambodian healthcare context, including AED availability.
It would have been helpful to know basic Khmer keywords, such as counting rhythm, “chest compression,” and “ventilation.”
Because I did not understand Khmer and was not confident in English, I could not be sure whether my explanations were fully understood, though I continued teaching.
Teaching in an environment where Japanese is not understood was a valuable experience.
I needed to consider how to teach BLS effectively in a foreign setting with different resources.
It would have been better if I could speak basic Khmer terms.
Language barriers were significant.
Some physicians understood English, but communication with non-medical participants was more difficult.
Time management was challenging because interpretation increased the duration to about 1.5 times that in Japan.
The instruction was difficult.
I felt a sense of accomplishment in spreading basic resuscitation skills to participants who had never received BLS training.
It was encouraging to see participants adapt BLS techniques to local needs during skill checks.
Communication skills were essential.
I needed to avoid relying entirely on the interpreter.
Communication was difficult.
Explaining technical skills was challenging.
It was my first experience teaching abroad.
Seeing participants enjoy learning despite language barriers gave me confidence.
Both verbal and non-verbal communication skills improved.
Khmer-language materials and AEDs are needed.
It was difficult to assess how much the participants actually understood.
Instructors must understand local medical needs and tailor the teaching accordingly.
I learned that teaching is still possible even when the language does not fully connect, which increased my confidence.

Selected responses from Japanese Association for Acute Medicine (JAAM)-certified Japanese instructors describing their experiences after teaching the basic life support (BLS) in Cambodia. Themes include language challenges, cultural reflection, learner engagement, and self-development.

#### 3.2.1 Communication and instruction

A prominent theme concerned instruction delivery through interpreters. The co-occurrence network demonstrated instructors frequently grappling with conveying concepts clearly, verifying comprehension, and adjusting explanations to linguistic constraints, highlighting the need for adaptive strategies.

#### 3.2.2 Learning processes and AED instruction

Another major cluster related to AED operational understanding and practical use. Network patterns suggested persistent challenges in explaining device operations, largely influenced by non-local language labels. Instructors expressed difficulty ensuring trainees could interpret prompts accurately and perform corresponding actions, reflecting equipment-language mismatch issues.

#### 3.2.3 BLS implementation and contextual adaptation

A third theme emerged around adjusting BLS instruction to resource-limited realities. This cluster highlighted instructors’ reflections on tailoring teaching methods, managing equipment constraints, and contextualizing BLS skills to local needs. The emphasis was on developing situationally appropriate teaching approaches in unfamiliar cultural and logistical settings.

### 3.3 Emotional tone

We determined that applying dictionary-based models (such as NRCLex) to free-form descriptions might not sufficiently reflect the context due to the short sentence structure of the translations and the lexical changes that occur during the translation process from Japanese to English. Therefore, this study did not use automatic analysis based on dictionaries. Instead, the research team manually classified the responses as “Positive,” “Negative,” or “Neutral” based on context (Table 3).

**TABLE 3:** Translated free-text comments from instructors.

Emotional tone	Subcategories (themes)	Representative statements (translated)
Positive	• Learner motivation • Cross-cultural interaction • Personal growth • Peer learning	“Participants were highly motivated.” “Cross-cultural interaction was meaningful.” “I gained confidence despite language barriers.” “Participants corrected each other during practice.”
Negative	• Language barriers • Interpreter-related difficulties • Limited Khmer-compatible materials • Difficulty assessing understanding • Logistical constraints	“It was unclear whether the interpreter conveyed the message accurately.” “Communication through an interpreter was difficult.” “Only one Khmer AED device was available.” “It was hard to judge whether participants understood.” “Transporting equipment through the airport was difficult.”
Neutral	• Need for English/Khmer proficiency • Need for localized materials • Adapting to local medical context • Recognition of instructional differences abroad	“Basic English and Khmer phrases are necessary.” “Teaching materials should be adapted to Khmer.” “Instruction must be tailored to local needs.” “Teaching abroad requires different preparation.”

This table summarizes all qualitative comments provided by the six Japanese instructors who participated in the basic life support (BLS) training program in Cambodia. The original responses were collected in Japanese and subsequently translated into English for analysis. Each comment reflects instructors’ perceptions related to communication challenges, cultural differences, instructional strategies, resource limitations, and personal growth during the training sessions.

The results of the classification showed that positive tones included “high learner motivation,” “the meaningfulness of intercultural exchange,” “self-growth through instruction,” and “collaborative learning among participants.” Conversely, negative tones were associated with “communication difficulties when using interpreters,” “shortage of Khmer-language materials and AEDs,” “difficulty gauging participants’ comprehension,” and “linguistic and instructional constraints.” Neutral tones included practical and descriptive comments such as “the necessity for basic communication in English and Khmer,” “optimizing materials for future training,” and “adjusting instructional content to local medical conditions.”

## 4 Discussion

This study analyzed Japanese BLS instructors’ experiences in an international course in Cambodia, integrating quantitative (VAS) and qualitative (text mining) data. While instructors reported high accomplishment and perceived educational significance, they recognized complex teaching challenges from language barriers and limited resources. These findings align with common difficulties in international BLS education in LMICs, highlighting cross-cultural educational practice characteristics.

Low VAS communication scores contrasted sharply with frequent use of terms like “communicate,” “understand,” and “teach” in open-ended comments, suggesting instructors experienced cognitive dissonance despite actively providing explanations due to uncertainty about content conveyance. This aligns with prior research highlighting uncertainty’s significant role in interpreter-mediated communication and language barriers in global health education (13, 14). Instructors needed strategies compensating for linguistic constraints, such as simpler terminology and non-verbal cues.

Resource scarcity posed significant educational challenges. Insufficient Khmer-labeled AED devices hindered participants’ comprehension and practical learning, aligning with systemic challenges in LMIC emergency education. Previous studies identified inadequate materials and equipment, lack of linguistic and cultural adaptation, and insufficiently standardized environments as factors limiting emergency education quality in LMICs (15, 16). Difficulty confirming understanding of practical skills like artificial respiration and AED operation aligns with relatively low VAS scores, reaffirming that verbal explanations and demonstrations are closely interrelated in practical skill instruction.

Conversely, instructors strongly experienced positive emotions—“fulfillment,” “growth,” and “confidence”—through educational activities. These emotions align with adult learning theory, cultural humility, and adaptive professionalism perspectives emphasizing that intercultural education requires reflective practice and flexible teaching strategies (6–8). Free-response comments revealed attitudes of adjusting teaching methods through trial and error under challenging circumstances, showing deepening educational perspectives through experience. Furthermore, instructors’ emotional and attitudinal growth is consistent with competency development models holistically considering knowledge, skills, and attitudes (KSA), suggesting cross-cultural environments foster instructors’ professional development (17). The KSA framework posits that developing cultural competence requires cognitive growth and maturation of affective and attitudinal aspects (18). Reflective and adaptive behaviors observed align with developmental processes supported by these frameworks.

During practical sessions, participants corrected each other and cooperated to practice skills. This emergent peer learning is significant because it extends beyond skill acquisition and contributes to regional capacity building (19). This phenomenon has been reported in BLS programs in other LMICs (20), suggesting that relationships where learners actively learn from each other could form foundations for sustainable educational systems in international BLS education.

Several educational implications emerge for designing future international BLS instructor training. First, preparatory training on communicating effectively through interpreters is essential where instructors and learners lack a common language. Second, culturally and linguistically adapted materials—including local-language AED prompts and simplified text—should be developed to improve clarity. Third, incorporating visual and non-verbal strategies may overcome linguistic constraints and enhance procedural skill comprehension. Furthermore, strengthening instructors’ understanding of local healthcare contexts, including available resources and post-resuscitation systems, is critical for context-appropriate training. Finally, international BLS programs should embed opportunities for reflective practice and adaptive teaching, enabling instructors to adjust approaches responding to cultural, linguistic, and resource-related challenges. These considerations highlight a pathway toward more sustainable and pedagogically grounded BLS education in LMICs.

## 5 Conclusion

This study examined Japanese instructors’ experiences teaching Basic Life Support (BLS) in Cambodia, highlighting educational challenges in cross-cultural and resource-limited environments. Despite facing linguistic barriers, lack of locally adapted materials, and difficulty gauging learners’ comprehension, instructors engaged in reflective learning and demonstrated adaptive teaching behaviors. Findings suggest that visual cues, simplified terminology, culturally and linguistically adapted materials, and effective interpreter collaboration are essential to international BLS training. Furthermore, understanding local healthcare contexts and integrating reflective and adaptive practice into instructor preparation may enhance sustainability and educational quality of future programs. This study provides initial insights informing the design of scalable, context-sensitive BLS education in LMICs.

## 6 Limitations

This study has several limitations. First, the sample size was small (*n* = 6), so the results should be interpreted as exploratory. It has been noted that small-scale qualitative research prioritizes contextual depth over generalization (21), and this study falls within that framework. Additionally, all VAS assessments and open-ended responses in this study are based on self-reported data, which can introduce biases inherent to self-reporting, such as social desirability or recall bias. Previous research has shown that common method bias can influence interpretations in behavioral science studies (22), and this possibility must be considered when interpreting the results of this study. Additionally, the free-response comments were translated from Japanese to English prior to analysis. It is known that translation in qualitative research can affect meaning and nuance (12). Although a review process was conducted after translation, some impact on vocabulary or frequency patterns cannot be ruled out. Additionally, due to the limited data volume, text mining involved manual emotion classification by the research team, which introduces potential subjectivity. Finally, Cambodia’s unique cultural and logistical context may not be directly applicable to other low- and middle-income countries (LMICs), which imposes limitations on the study’s external validity.

## Data Availability

The original contributions presented in this study are included in this article/Supplementary material, further inquiries can be directed to the corresponding author.

## Supplementary material

Supplementary Table 1 Course timetable. Timetable for the JAAM-certified basic life support (BLS) course conducted in Cambodia. The session consisted of an introduction, core CPR and AED training, and a wrap-up. Each session lasted 150 min.
