# Supplementary figures and images for: Evaluation of Japanese instructor experiences in the first overseas BLS course certified by the Japanese association for acute medicine in Cambodia: a mixed-methods analysis using text mining
Source: Front Med (Lausanne). 2026 Jan 5;12:1679781. doi: 10.3389/fmed.2025.1679781 (PMC12812561; doi:10.3389/fmed.2025.1679781)

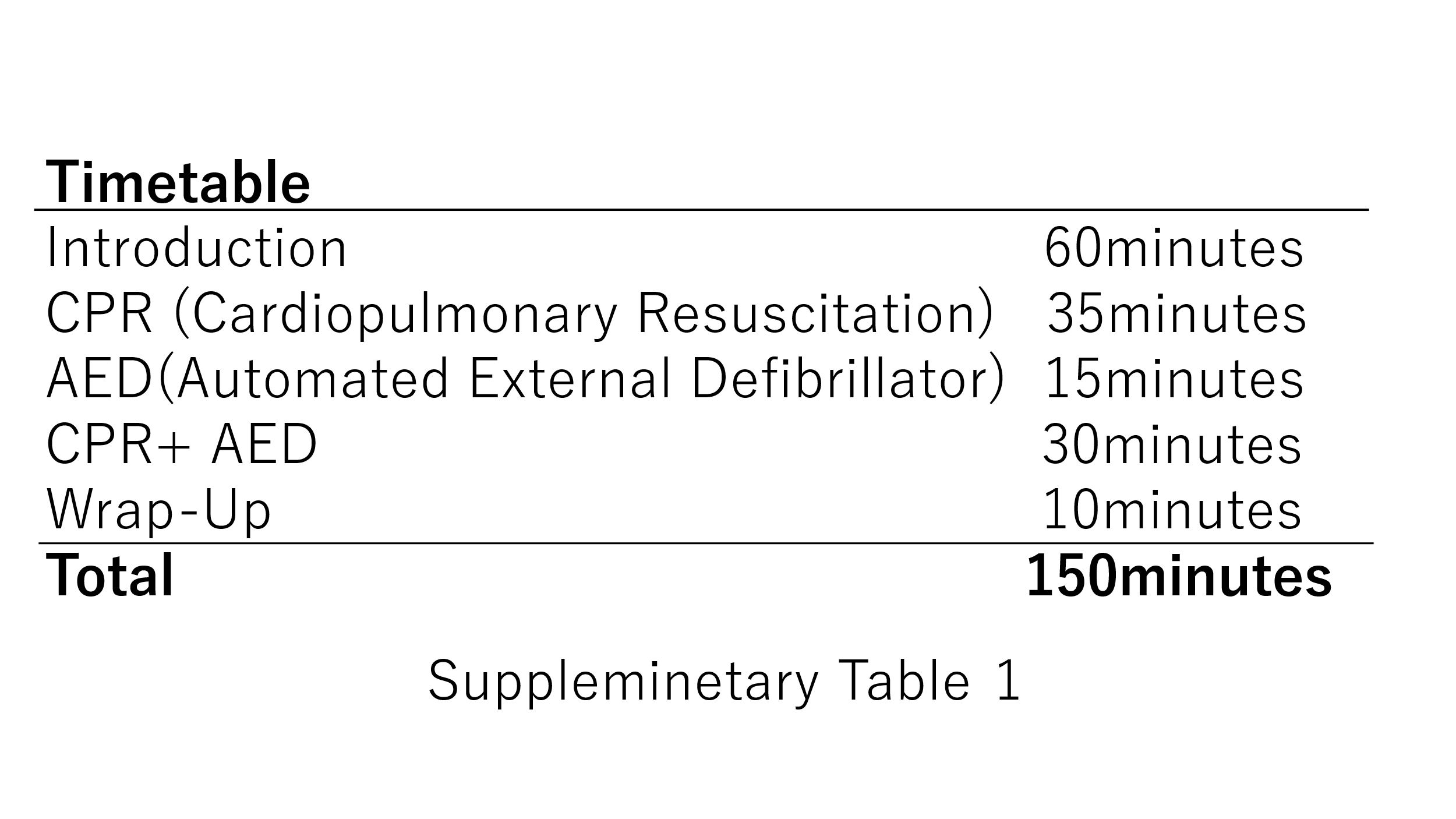

Supplement: Supplementary Table 1 — Course timetable. Timetable for the JAAM-certified basic life support (BLS) course conducted in Cambodia. The session consisted of an introduction, core CPR and AED training, and a wrap-up. Each session lasted 150 min. [file Table_1.docx]
